# A Digital Twin Framework for Per-Fraction Prediction of Normal Tissue Toxicity in Radiotherapy of Non-Small Cell Lung Cancer

**DOI:** 10.3390/jpm16100495

**Published:** 2026-09-24

**Authors:** Anvi Sud, Jialu Huang, Gregory R. Hart, John Kim, Lauren Tressel, Henry S. Park, Jun Deng

**Affiliations:** 1 Department of Biomedical Informatics and Data Science, Yale University, New Haven, CT 06520, USA; anvi.sud@yale.edu; 2 Department of Statistics and Data Science, Yale University, New Haven, CT 06520, USA; jialu.huang@yale.edu; 3 Division of Natural Sciences, University of Guam, Mangilao, GU 96923, USA; gregory.hart@triton.uog.edu; 4 Department of Therapeutic Radiology, Yale University, New Haven, CT 06510, USA; john.kim@ynhh.org (J.K.); lauren.tressel@ynhh.org (L.T.); henry.park@yale.edu (H.S.P.)

**Keywords:** digital twins, non-small cell lung cancer, personalized radiotherapy, multimodal deep learning, clinical decision support

## Abstract

**Background/Objectives:** Stereotactic body radiation therapy (SBRT), often delivered in 3–5 fractions, is a highly effective treatment strategy for early-stage non-small cell lung cancer (NSCLC) in medically inoperable patients. Biology-guided radiotherapy (BGRT), a PET-guided SBRT, enables acquisition of patient-specific metabolic, anatomical, and dose information at each fraction. While prior models have focused on static, population-level prediction of radiation-induced toxicity, there remains a lack of approaches to characterize how normal tissue response evolves longitudinally within individual patients over the course of radiation treatment. We therefore hypothesize that AI-based models can quantify temporal toxicity patterns in such longitudinal data, allowing clinicians to balance tumor control against normal tissue complications to guide individualized treatment planning. **Methods:** In this proof-of-concept study, we developed COMPASS (COMprehensive Personalized Assessment System), a digital twin framework that generates per-fraction risk estimates that could inform future adaptive decision-making in NSCLC. With Yale Institutional Review Board approval, eight patients with NSCLC who underwent BGRT were retrospectively included in this study. A total of 99 organ–fraction observations, spanning 24 patient–organ longitudinal trajectories across three organs-at-risk (spinal cord, heart, and esophagus), were used to train a Gated Recurrent Unit (GRU) autoencoder to learn latent temporal representations, which were subsequently characterized using logistic regression to assess for CTCAE ≥ 1 toxicity. **Results:** In a leave-one-out patient evaluation, COMPASS achieved an area under the curve (AUC) of 0.90, with 80% sensitivity and 78.6% specificity in predicting normal tissue toxicity at the patient–organ trajectory level on a fractional basis. **Conclusions:** These findings support the feasibility of AI-driven digital twins for per-fraction toxicity monitoring, offering a step toward personalized radiotherapy for NSCLC patients.

## 1. Introduction

The standard of care for early-stage non-small cell lung cancer (NSCLC) is surgical resection via minimally invasive techniques with mediastinal lymph node evaluation [1]. For medically inoperable patients, stereotactic body radiation therapy (SBRT), often delivered in 3–5 fractions, is a highly effective alternative [2]. SBRT of NSCLC represents a core therapeutic dilemma: escalating tumor dose improves local tumor control but increases the risk of severe toxicity to adjacent critical structures such as the heart, lungs, esophagus and spinal cord [3]. Clinically speaking, accurate and prompt prediction of normal tissue toxicity in radiotherapy of NSCLC can reduce severe treatment-related complications, improve quality of life, enable safer dose escalation, support individualized treatment decisions, improve treatment adherence, lower hospitalization rates, and potentially improve long-term survival. Current clinical practices rely on population-derived normal tissue complication probability (NTCP) models and dose–volume histogram (DVH) constraints to evaluate toxicity risk during treatment planning [4]. While these techniques have guided safe therapy for decades, they face well recognized limitations in the era of adaptive radiation therapy (ART) and patient-specific modeling approaches. Anatomical deformation, organ mobility, tumor regression, and the evolving biological response that occurs during fractionated delivery cannot be accounted for through static treatment evaluations based on planning CT [5]. Standard DVH metrics, while capturing volumetric dose distributions, aggregate dose across entire organs without preserving spatial heterogeneity or integrating voxel-wise biological markers that reflect early injury signals [6]. Above all, these methodologies do not provide a concrete mechanism for updating risk predictions in real time as new imaging becomes available at each treatment fraction, thereby limiting the possibility of an adaptive intervention before irrevocable toxicity becomes clinically apparent. These limitations motivate approaches capable of generating and continuously updating patient-specific risk estimates using longitudinal, per-fraction data throughout the course of treatment.

Recently, biology-guided radiotherapy (BGRT) has incorporated positron emission tomography (PET) imaging into treatment delivery which enables repeated acquisition of functional and anatomical information at each fraction, thereby facilitating longitudinal assessment of treatment response (as shown in Figure 1) [7]. These time series datasets contain patient-specific trajectories of dose accumulation and imaging-derived changes, including radiomic feature evolution and spatially heterogeneous dose distributions [8]. The clinical values of PET and radiomic features have also been explored in other radiotherapy settings, including recurrent high-grade glioma, where such features have been investigated for distinguishing progression from pseudoprogression [9]. As radiomics, genomics, and AI-based models mature, toxicity prediction is increasingly moving from population-level risk estimation toward truly patient-specific risk assessment. This data may also allow for the characterization of both localized areas of enhanced sensitivity and organ-level response patterns when analyzed over time. By integrating voxel-level biologically equivalent dose (BED) accumulation with spatial dose heterogeneity and imaging-derived features, it becomes possible to explore spatially resolved patterns that may precede clinically apparent injury [10]. However, these relationships remain largely under-explored.

In this proof-of-concept study, we present COMPASS (COMprehensive Personalized ASsessment System), a digital twin framework intended to model normal tissue response as a temporal process specific to each patient during radiotherapy. Our approach blends unsupervised representation learning through GRU (Gated Recurrent Unit) autoencoding, enabling extraction of compact temporal embeddings that reflect each organ’s biological trajectory followed by a supervised toxicity classification [11]. This extends established machine learning frameworks in radiation oncology for outcome prediction by incorporating dynamic temporal modeling of per-fraction data [12].

Rather than presenting a fully realized digital twin or demonstrating superiority over existing clinical models, this work investigates two focused questions: whether dense per-fraction multimodal phenotyping captures patient-specific dose–response patterns predictive of eventual toxicity, and whether individual patient–organ trajectories contain biological signals that discriminate toxic from non-toxic outcomes.

## 2. Materials and Methods

### 2.1. Patient Cohort and Data Acquisition

With institutional ethical review waived under Yale Human Research Protection Program Exempt Category 4 (iii) (Protocol #2000042387), eight patients with early-stage non-small cell lung cancer receiving definitive fractionated radiotherapy were retrospectively included in this proof-of-concept study. Inclusion criteria consisted of patients treated on Reflexion (RefleXion, Hayward, CA, USA), a PET-enabled radiotherapy platform for BGRT, with available per-fraction PET and kilovoltage computed tomography (kVCT) imaging, complete treatment planning data, and follow-up toxicity assessment [7]. Consistent with BGRT clinical practice, eligible patients had (fluorodeoxyglucose) FDG-avid tumors that were well visualized on PET imaging and suitable for PET-guided targeting, with anatomical and motion characteristics compatible with reliable image-guided delivery. Cases were selected where tumor localization and separation from adjacent critical structures allowed for clinically acceptable treatment planning and organ-at-risk sparing within a hypo-fractionated SBRT framework. Data was collected from 27 August 2024 to 6 November 2025. Treatment regimens ranged from 3 to 5 fractions per patient, generating 99 total organ–fraction observations across 24 distinct individual patient–organ trajectories (heart, esophagus, spinal cord). PET and kVCT imaging were acquired at each treatment fraction to provide in-treatment biologic localization of metabolically active tumor regions. A treatment planning CT was performed prior to treatment to generate a high-resolution three-dimensional anatomical rendering for target delineation and treatment planning. Planned and delivered dose distributions were computed for each fraction, enabling longitudinal tracking of spatial dose deposition across the course of radiation treatment.

From every in-person and remote consultation, patient-reported outcomes were extracted along the time for each patient 6–11 months post-BGRT from our cloud-based radiation oncology information system Aria. Toxicity outcomes were graded contemporaneously at each clinical encounter during follow-up according to the Common Terminology Criteria for Adverse Events (CTCAE), version 5.0, as part of routine clinical care. These retrospectively recorded CTCAE grades were subsequently abstracted from the medical records for this analysis [13]. With 10 out of 24 patient–organ trajectories exhibiting toxicity, binary classification categorized results as Grade 0 (no toxicity) against Grade ≥ 1 (any evident harm). This ratio reflected the high-risk nature of thoracic radiotherapy near dose-limiting organs while providing sufficient outcome diversity for supervised learning, but this endpoint recompensed mild events that may not be clinically consequential or treatment limiting.

For clarity, we define the hierarchical structure of the dataset as follows. The patient (*n* = 8) is the independent sampling unit. The main unit of prediction and evaluation is the 24 patient–organ trajectories that result from each patient contributing observations from three organs-at-risk; that is, the model generates one final toxicity probability for each patient–organ pair. The temporal model uses the three to five fraction-level observations (a total of 99) that make up each trajectory as time series inputs. At the patient–organ trajectory level, performance measures (AUC, sensitivity, specificity, accuracy, and Brier score) are calculated using 24 test cases combined over 8 leave-one-patient-out (LOPO) folds. Fraction-level predictions are intermediate outputs used to illustrate longitudinal risk evolution and are not themselves the unit of performance evaluation.

### 2.2. Data Preprocessing and Region of Interest (ROI) Delineation

To ensure consistency across the integration of multimodal features at the voxel level, we implemented a standardized preprocessing pipeline that co-registered PET, CT, DICOM RTDose, and DICOM RTSTRUCT data into a single, spatial reference frame. This processing was performed utilizing core libraries including pydicom (version 2.4.4) for DICOM parsing and SimpleITK (2.5.0) for image manipulation and registration, a combination of tools recognized for constructing robust medical image processing workflows [14,15].

For each patient, the time-averaged Average Intensity Projection (AIP) CT scan (as shown in Figure 2), a 3D image derived from the pre-treatment 4DCT acquired during simulation, served as the spatial reference frame for all longitudinal data and represented the voxel-wise average intensity across the patient’s breathing cycles [16].

The AIP CT was reconstructed using rt-utils and exported as a NIfTI volume (typical size: 512 × 512 × 175 voxels). Regions of interest (ROIs) for the heart, esophagus, and spinal cord were generated by parsing the contours from DICOM RTSTRUCT files and converting DICOM contours into 3D binary masks, which were also subsequently saved in NIfTI format [17].

To ensure that ROI masks and the AIP reference frame were co-registered, geometric transformations obtained from DICOM metadata were used. To extract synchronized radiomic, dosiomic, and temporal features from per-fraction PET and kVCT images, delivered dose distributions, and cumulative BED maps across all treatment fractions, this AIP-aligned dataset offered a consistent voxel-wise coordinate system for integrating longitudinal data [18,19].

### 2.3. Multimodal Feature Engineering

Using a voxel-wise biological dose computation pipeline intended to maintain spatial dose variability, we retrieved 73 characteristics from seven complementary modalities (shown in Figure 3) for every patient–organ–fraction observation (as shown in Table 1). We first computed per-voxel biological effective dose (BED) for each fraction using the linear–quadratic model BED = d × (1 + d/(α/β)), where d is the physical dose per voxel and α/β was organ-specific (3 Gy for heart and esophagus and 2 Gy for spinal cord) [20]. Voxel-wise BED values were then summed across fractions to yield cumulative BED voxel-wise accounting for the nonlinear dose–response where high-dose voxels contribute disproportionately to biological effect. This cumulative voxel-wise BED distribution was converted to equivalent dose in 2-Gy fractions (EQD2) via EQD2 = BED/(1 + 2/(α/β)), preserving spatial heterogeneity before feature aggregation.

From these voxel-wise EQD2 distributions, we extracted traditional dosimetric features comprising dose statistics (Dmax, Dmean, Dmin, D5, D50, D95), volume-threshold metrics (D2cc, V5, V10, V20), and plan quality indices (dose homogeneity, conformity, and gradient indices) [21,22]. Critically, dose distribution features performed spatial analysis of per-fraction physical dose distributions (prior to cumulative summation), extracting percentile doses (Dxp quantifying dose to top x% of voxels), volume-percentile metrics (VxMax indicating percentage receiving > x% of maximum dose), and distribution shape parameters (skewness, kurtosis) [23,24]. These per-fraction dosiomics capture intrafraction dose heterogeneity patterns, while cumulative EQD2 features quantify total biological burden; together, they provide complementary temporal (per-fraction patterns) and cumulative (biological endpoint-relevant) dose characterization. The percentile-based dosiomics demonstrated more sensitivity to focal hotspots compared to traditional volume thresholds in the results; for example, spatially localized high-dose regions may be masked by global volume thresholds (e.g., V10), motivating voxel-level or percentile-based descriptors for characterizing heterogeneous dose distributions in serial organs such as the spinal cord [25].

Temporal kinetics features quantified dose accumulation dynamics through changes in cumulative mean EQD2 per fraction (ΔDmean_EQD2), derived using linear–quadratic models, along with inter-fraction time intervals to capture potential recovery effects between treatments. Organ-level geometric features included total organ volume and fractional hotspot burden, defined as the percentage of volume receiving > 80% of the maximum dose, consistent with established DVH-based dosimetric evaluation metrics [21].

CT and PET radiomic features were extracted from voxel intensity distributions and included first-order histogram statistics (mean, standard deviation, minimum, maximum, median) and higher-order distributional features such as skewness and kurtosis [26]. Texture-related metrics, including entropy and uniformity, were also computed.

Feature preprocessing included Spearman correlation filtering (ρ > 0.90), median imputation, and Z-score normalization, applied solely within training folds only to avoid data leakage in leave-one-patient-out cross-validation [27].

### 2.4. Temporal Sequence Modeling Architecture

The key aspect of COMPASS is that it models each patient–organ pair as a sequence across treatment fractions, enabling analysis of how dose–response evolves over time rather than treating fractions as independent observations (as shown in Figure 4). Grouping observations by (patient, organ) and sorting by fraction index resulted in sequences with different lengths (3–5 timepoints), depending on the treatment schedule. The multimodal feature vector at that fraction is contained in each sequence element, resulting in input tensors of shape (N_sequences, max_fractions, n_features), where N_sequences is the number of distinct patient–organ pairs.

We implemented a recurrent autoencoder using Gated Recurrent Units (GRUs) to learn compressed latent representations of these temporal trajectories [28]. The encoder comprised two stacked GRU layers: the first layer (input_dim → 16 hidden units, dropout = 0.5) processed the full feature sequence, and the second layer (16 → 8 hidden units) produced the final latent embedding by extracting the final hidden state. Hidden-layer sizes were fixed prior to cross-validation to keep representational capacity modest relative to the limited number of training sequences per fold.

Given the dataset size, the GRU architecture was chosen over LSTM due to its computational efficiency and lower parameter count, while keeping the gating mechanisms suited to capture long-range temporal relationships in dosage accumulation. The decoder reconstructs the original sequence from the compressed latent representation. The 8-dimensional latent vector is first transformed through a learned initialization layer to produce the decoder’s initial hidden state (8 → 16 units via tanh activation). This latent vector is then repeated across all timesteps and processed through a GRU layer (16 hidden units) initialized with the learned hidden state. Finally, a linear projection layer maps the GRU outputs back to the original feature dimensionality (16 → input_dim), reconstructing the dose–response trajectory. Mean squared error between input and reconstructed sequences, masked to account for variable sequence lengths, served as the unsupervised training objective. The model was trained using Adam optimization with weight decay regularization (λ = 1 × 10^−4^) and gradient clipping (max_norm = 1.0) to ensure stable convergence over 160 epochs with learning rate 0.0005, using mini-batch gradient descent with batch size 4 and sequences shuffled each epoch. Reconstruction loss was monitored throughout training as a convergence diagnostic; because only the trained encoder’s latent embeddings, not the decoder, are used for downstream toxicity classification.

The autoencoder’s 8-dimensional latent embedding provides a condensed representation of each patient–organ’s trajectory, capturing baseline PET-derived features, temporal patterns of dose accumulation and imaging-derived changes, and organ-specific variation observed across the dataset. In contrast to static feature vectors, this approach allows for the definition of how model-derived risk estimations may change over time by incorporating temporal information across fractions.

### 2.5. Supervised Toxicity Prediction

The autoencoder’s learnt embeddings were directly used for toxicity prediction. The 8-dimensional latent space was mapped to the eventual toxicity probability (binary outcome: Grade ≥ 1 vs. Grade 0) using a logistic regression classifier with L2 regularization and balanced class weights. Given the relatively small sample size (24 patient–organ pairs over 7 training patients per fold), logistic regression was purposefully selected over neural network classifiers to reduce the likelihood of overfitting. The two-stage approach unsupervised representation learning followed by supervised classification enables incorporation of temporal patterns captured in the embeddings while maintaining interpretable probabilistic outputs.

The model produces two related but distinct outputs. The final-fraction prediction is generated by encoding the complete observed patient–organ sequence through all delivered fractions into a single latent embedding, which the classifier then scores. During ongoing treatment, the model additionally generates per-fraction predictions: at each fraction t, the partial sequence observed so far (fractions 1 through t) is encoded into its own latent embedding and scored by the classifier, producing a distinct toxicity probability P (toxicity|cumulative features_1−t_) at every fraction as treatment progresses, rather than a single probability computed only once at the end of the sequence. This allows risk estimates to be updated as new data arrives. This sequential prediction capability distinguishes our prototype from static pre-treatment models and enables adaptive clinical decision-making. The autoencoder also computes reconstruction error for each sequence as a secondary quality metric, which can flag unusual dose–response patterns that deviate from training data, though the primary model outputs are the supervised toxicity probabilities.

### 2.6. Cross-Validation Strategy

Leave-one-patient-out (LOPO) cross-validation was used to evaluate model performance on completely unseen patients [29]. In each of the eight folds, one patient was held out entirely while the remaining seven patients’ data was used for training the autoencoder, fitting the supervised classifier, and tuning all hyperparameters. This design ensured that the test patient’s biological trajectory across all fractions and organs is completely unseen during model development to approximate evaluation for a completely unseen patient.

Specifically, the following steps were performed exclusively on the seven training patients within each fold: (1) Feature selection: Spearman correlation-based redundancy removal (threshold ρ > 0.90) was computed on training features only. The resulting feature subset was then applied to the test patient. (2) Feature imputation: median values for imputation were computed from training patients only using scikit-learn SimpleImputer; test-patient missing values were imputed using these training-fold medians. (3) Feature standardization: Z-score parameters (mean, standard deviation) were estimated from training patients only using StandardScaler; the same parameters were then applied to transform the test patient. (4) GRU autoencoder training: the autoencoder was trained on training-fold sequences only and was not exposed to any data from the test patient prior to encoding. (5) Logistic regression fitting: the supervised classifier was fit on the latent embeddings derived from training patients only. (6) Hyperparameter selection: architecture choices (GRU layer sizes, dropout rate, learning rate, regularization) were fixed a priori and not tuned within each fold to avoid optimization-based leakage. (7) Aggregation strategy: the final-fraction prediction is generated by encoding the complete observed sequence through the last delivered fraction into a single latent embedding, which the classifier then scores. Per-fraction predictions are generated separately at each intermediate fraction, from the partial sequence observed up to that point, and are retained only to characterize the longitudinal evolution of estimated risk rather than for the primary evaluation, which is done using the final fraction against the eventual toxicity outcome. (8) Operating-point threshold selection: following model evaluation, organ-specific probability thresholds were selected from the observed predictions to identify operating points that prioritized sensitivity for toxicity classification. The selected thresholds were 0.4 for esophagus, 0.6 for heart, and 0.5 for spinal cord. These thresholds were then fixed for the calculation of sensitivity, specificity, accuracy and construction of the corresponding confusion matrix. The eventual CTCAE toxicity outcome recorded during clinical follow-up was used as the ground-truth endpoint for evaluating model predictions.

## 3. Results

### 3.1. Performance Metrics

Model performance was evaluated using leave-one-patient-out (LOPO) cross-validation [29,30]. The primary unit of evaluation was the patient–organ trajectory, with metrics computed across 24 test instances and averaged across folds. COMPASS achieved AUC 0.907 (95% CI: 0.793–1.000), sensitivity 80.0% (95% CI: 40.0–100.0%), specificity 78.6% (95% CI: 53.8–94.7%), accuracy 79.2% (95% CI: 62.5–95.8%), and Brier score 0.140 (95% CI: 0.065–0.216) across eight LOPO folds. Confidence intervals were estimated by patient-level bootstrap resampling (*n* = 5000 iterations): at each iteration, eight patients were drawn with replacement, and all three organ trajectories of each sampled patient were included, yielding 24 instances per resample and preserving the within-patient correlation structure of the LOPO design. The wide intervals reflect the small number of independent patients (*n* = 8) and should be interpreted as preliminary proof-of-concept estimates rather than stable population-level benchmarks. Mean training-side AUC across the eight LOPO folds was 0.906 (range 0.706–1.000, median 0.958).

Discrimination was quantified using the area under the receiver operating characteristic curve (AUC-ROC), reflecting the model’s ability to rank patient–organ pairs by toxicity risk. The Brier score was used as a measure of overall probabilistic accuracy, computed as the mean squared error between predicted probabilities and observed outcomes [31].

Classification performance was further characterized using the organ-specific probability thresholds selected to prioritize sensitivity (esophagus = 0.4, heart = 0.6, spinal cord = 0.5). Sensitivity, specificity, and accuracy at these operating points were 80.0%, 78.6%, and 79.2%, respectively, and are reported as apparent operating-point metrics. The corresponding confusion matrix summarizes the 24 unique patient–organ test cases generated across the eight LOPO folds (Figure 5).

### 3.2. Predictive Performance and Early Warning Capability

Predictions generated at the final treatment fraction, using all accumulated data from earlier fractions, were aggregated because toxicity outcomes were assessed at follow-up after treatment completion, making these estimates directly comparable to the observed outcome labels. In this proof-of-concept setting with binary eventual toxicity labels, thresholding this probability reflected the clinical question of whether a given organ will develop toxicity by the end of treatment for smooth evaluation. Per-fraction risk scores were also generated after each delivered fraction to enable sequential assessment of evolving toxicity risk over the course of treatment. The model’s early warning capability is demonstrated as well (as shown in Figure 6), where patient–organ trajectories that ultimately developed CTCAE ≥ 1 toxicity show an overall increase in predicted risk across fractions, whereas non-toxic trajectories remain low and stable. The accompanying table indicates cases where predictions did not align with observed outcomes.

## 4. Discussion

This study shows that time series per-fraction multimodal features can support early prediction of normal tissue toxicity by providing additional predictive signals beyond conventional dose-based metrics. In this cohort, the patient–organ longitudinal risk trajectories consistently showed a difference in toxic versus non-toxic cases, with toxic cases exhibiting increasing risk during treatment while non-toxic cases remained low and stable.

Prior studies in normal tissue toxicity prediction have explored imaging- and dose-based prediction of radiation toxicity using radiomics, multimodal fusion approaches, dosiomics, 3D deep learning frameworks, and longitudinal modeling strategies to inform adaptive replanning [32,33,34]. COMPASS builds upon these approaches by incorporating sequential per-fraction multimodal phenotyping and generating continuously updated risk estimates throughout treatment. However, the small cohort size and inter-patient variability limit the strength of any translational conclusions.

### 4.1. Potential for Inter-Fractional Intervention

An example from patient 6′s esophageal trajectory illustrates how COMPASS captures temporal imaging and dosimetric changes not fully reflected in standard dose summaries. At fraction 3 of 5, the model estimated elevated toxicity risk despite cumulative esophageal dose remaining below planning constraints. This divergence was accompanied by increased PETmean relative to baseline and emerging focal dose heterogeneity, reflecting measurable changes in PET-derived intensity and spatial dose distribution during treatment. Here, PETmean refers to a first-order PET intensity feature derived from voxel-wise, standardized uptake values, representing a quantitative imaging signal without implying biological interpretation [35,36].

Patient 3′s cardiac trajectory similarly demonstrates progressively increasing risk probabilities across fractions (0.830 → 0.956 → 0.969 → 0.970 → 0.971), alongside dosimetric evidence of heterogeneous exposure, including D98p = 0.54 Gy, D2p = 28.59 Gy, and V5 = 55.4%, indicating spatially non-uniform dose distribution with focal high-dose regions. These signals represent multimodal imaging and dosimetric descriptors within the COMPASS framework and should be interpreted strictly as correlates of potential response patterns rather than evidence of tissue injury or clinically manifest toxicity.

In contrast, patient 1′s non-toxic trajectories maintained stable risk estimates and imaging features throughout treatment, including consistently low probabilities (<0.30) and minimal dose heterogeneity.

Overall, COMPASS integrates longitudinal PET-derived intensity features, CT radiomic variation, and spatial dose heterogeneity to generate sequential risk estimates across treatment fractions. These components represent complementary quantitative descriptors of evolving treatment response, while clinically relevant toxicity remains defined exclusively by observed clinical outcomes rather than intermediate imaging or dosimetric signals.

### 4.2. Limitations

There are a few limitations that warrant this discussion. While LOPO cross-validation helps reduce the risk of overfitting and prevents data leakage, there are only eight patients in the cohort, which limits generalizability and makes conducting thorough subgroup analysis by organ, dosing schedule, or toxicity mechanism difficult. Validation in larger, independent, multi-institutional cohorts will be necessary to ascertain whether the paradigm is transferable across imaging techniques, treatment modalities, and patient populations.

Second, the CTCAE ≥ 1 (any detectable toxicity) was selected as the classification endpoint for two reasons. First, as an exploratory proof-of-concept study, this threshold maximizes the number of positive cases available for learning (10/24 trajectories), which is essential in a small cohort. Second, the COMPASS framework is intended as longitudinal per-fraction toxicity risk surveillance framework; detecting Grade 1 signals may provide earlier warning before progression to higher-grade, clinically irreversible injury. We acknowledge that Grade 1 toxicities are often transient and of limited direct clinical consequence. Validation against CTCAE ≥ 2 endpoints, which represent toxicities more consistently requiring clinical intervention, is an important future direction but was precluded here by the small number of Grade ≥ 2 cases in this cohort.

Third, the current framework predicts future toxicity but does not predict the exact moment of toxicity onset. Because the labels are binary at the patient–organ level, all fractions receive the same outcome label regardless of when symptoms manifest. This limits differentiation between toxicity that arises during therapy and toxicity that manifests after treatment is completed. Access to larger cohorts with time-to-event outcomes or repeated toxicity assessments would enable more temporally precise modeling.

### 4.3. Translational Considerations and Implementation Challenges

While COMPASS demonstrates promising proof-of-concept performance, practical implementation barriers remain before clinical deployment can be considered. Feature extraction from per-fraction PET/CT, DICOM RTDose, and RTSTRUCT files was completed in approximately 15–25 min on a standard research laptop (Apple M4, no GPU), with GRU encoding and risk prediction adding under 2 min with a total turnaround of 17–27 min. In the BGRT workflow, each fraction takes 75–80 min including 45 min of PET radiotracer uptake, 5 min of tumor localization via kVCT and PET scans, and 25–30 min of radiation treatment delivery. There will be at least 24 h between two fractions of BGRT treatment where clinical intervention is possible if deemed necessary. Segmentation reproducibility was not formally assessed; the pipeline relies on expert-generated RTSTRUCT contours, and contour variability is a known source of radiomic and dosiomic feature uncertainty that would need to be characterized before deployment. Workflow integration would require automated DICOM export from the treatment planning system, a dedicated automatic feature extraction service, and a clinician-facing risk dashboard alongside software validation and regulatory consideration as a clinical decision support tool. Interpretability is currently limited to predicted probabilities from logistic regression on GRU latent embeddings; more detailed feature attribution methods are planned for future work. Taken together, COMPASS offers a possibility for inter-fractional intervention if clinically justified. Future implementation could further streamline the workflow by leveraging imaging already acquired during BGRT and optimizing the timing and processing of this data, supporting efficient per-fraction assessment while maintaining patient comfort. However, more prospective workflow integration and validation studies would be needed for seamless integration into a real-world radiotherapy workflow.

### 4.4. Future Directions

These limitations motivate several directions for future development of the COMPASS framework for clinical actionability, including expanding to larger, multi-institutional cohorts to improve generalizability and enable subgroup analyses across organs, dosing schedules, and toxicity mechanisms.

A next step is also to incorporate the effect of tumor control and provide a balanced risk score with a real-time dashboard for a comparative study: patients assigned to standard-of-care versus COMPASS-guided adaptive therapy, with toxicity reduction as the primary endpoint. Does adaptive re-planning maintain tumor control while preventing toxicity? We plan on using the reaction-diffusion equation to model tumor growth with the linear–quadratic model for tumor response to treatment. We will use a Bayesian framework to allow us to capture uncertainty and update our predictions with each fraction and its accompanying images/data.

Broader implications extend beyond radiotherapy. The core principles of dense longitudinal phenotyping, temporal sequence modeling, and patient-specific trajectories apply wherever treatment response evolves over time and biological heterogeneity confounds population predictions. Chemotherapy monitoring could track cumulative cardiotoxicity, immunotherapy surveillance could predict immune-related adverse events, and surgical outcomes could model post-operative complication risk. The key conceptual shift is recognizing that, for temporal processes, depth of within-patient observation can compensate for breadth of between-patient sampling.

Our research also aims to include per-voxel probability heatmaps derived from physics-based thresholds, extending toxicity assessment beyond uniform organ-level risk estimation to a spatially resolved framework (as shown in Figure 7). These maps would reveal that organs have different radiation tolerances, with patient-specific subregions being more vulnerable at doses considered safe under population-averaged restrictions. This technique, which projects voxel-wise risk directly onto anatomical representations, offers clinicians actionable spatial information regarding differential dose tolerance inside and across organs, enabling more informed decision-making. Specifically, it supports recognition that distinct organ subregions may fail at different dose thresholds, thereby reframing toxicity prediction from a binary organ-level outcome to a spatially informed risk landscape that aligns with clinical reasoning for dose escalation, selective sparing, and adaptive replanning.

## 5. Conclusions

We introduce COMPASS, a digital twin framework that uses longitudinal, multimodal data to investigate patient-specific temporal trends in radiation-induced response. By combining per-fraction PET and kVCT imaging, dosimetric features, and GRU-based representations within a leave-one-patient-out evaluation setting, we demonstrate the feasibility of learning trajectory-based representations of normal tissue response.

Within this cohort, the model captured temporal variation in dose–response patterns and generated evolving per-fraction risk estimates during treatment. The primary contribution of this study is therefore not a clinically validated predictive model, but a demonstration that longitudinal, per-fraction data can be incorporated into a patient-specific modeling framework. Future work will require larger cohorts with clinically meaningful endpoints (e.g., Grade ≥ 2 toxicity), as well as prospective validation, to determine whether such approaches provide added value beyond conventional dose-based models.

## Figures and Tables

**Figure 1:**
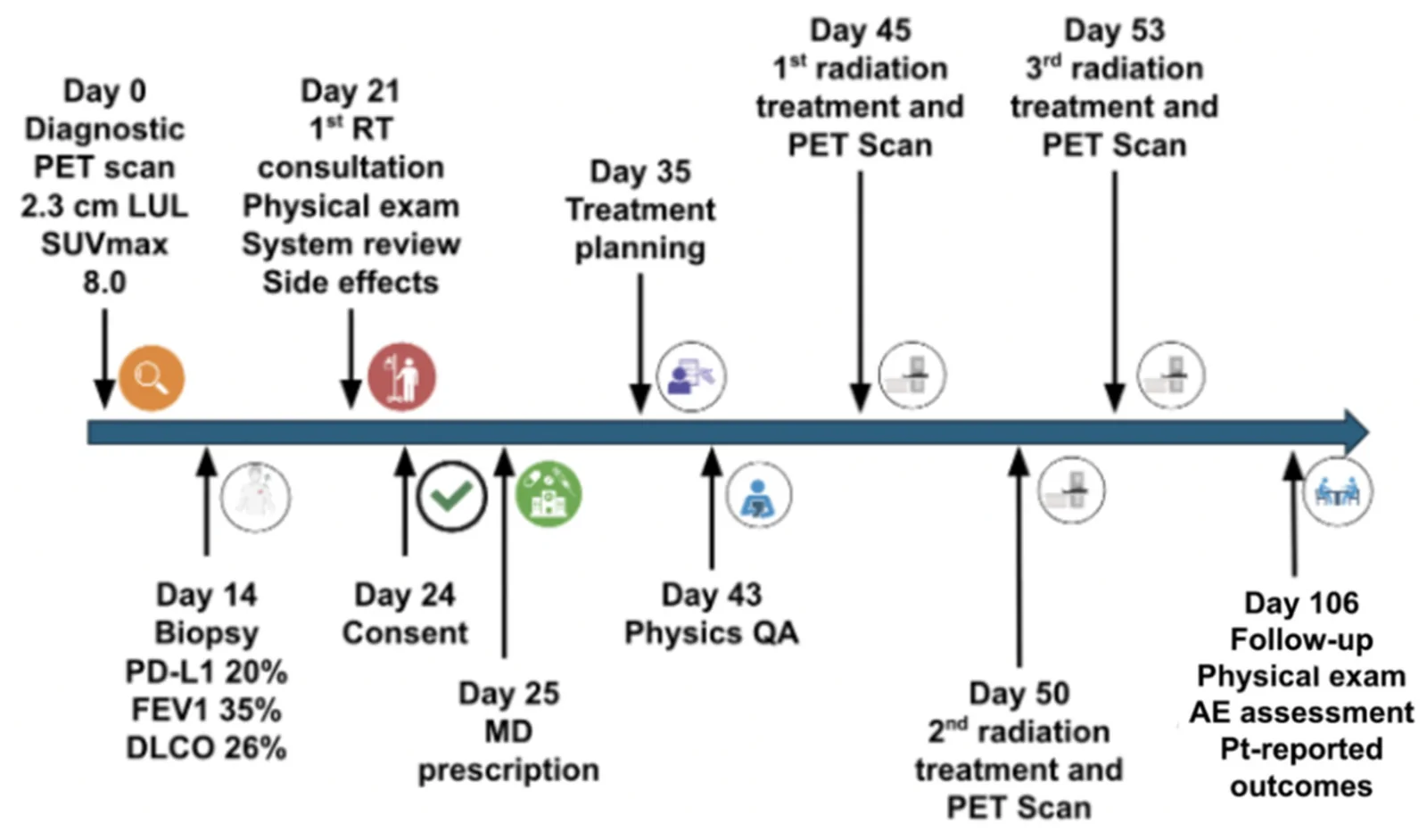
A typical clinical workflow for non-small cell lung cancer patients undergoing biology-guided radiation therapy (BGRT).

**Figure 2:**
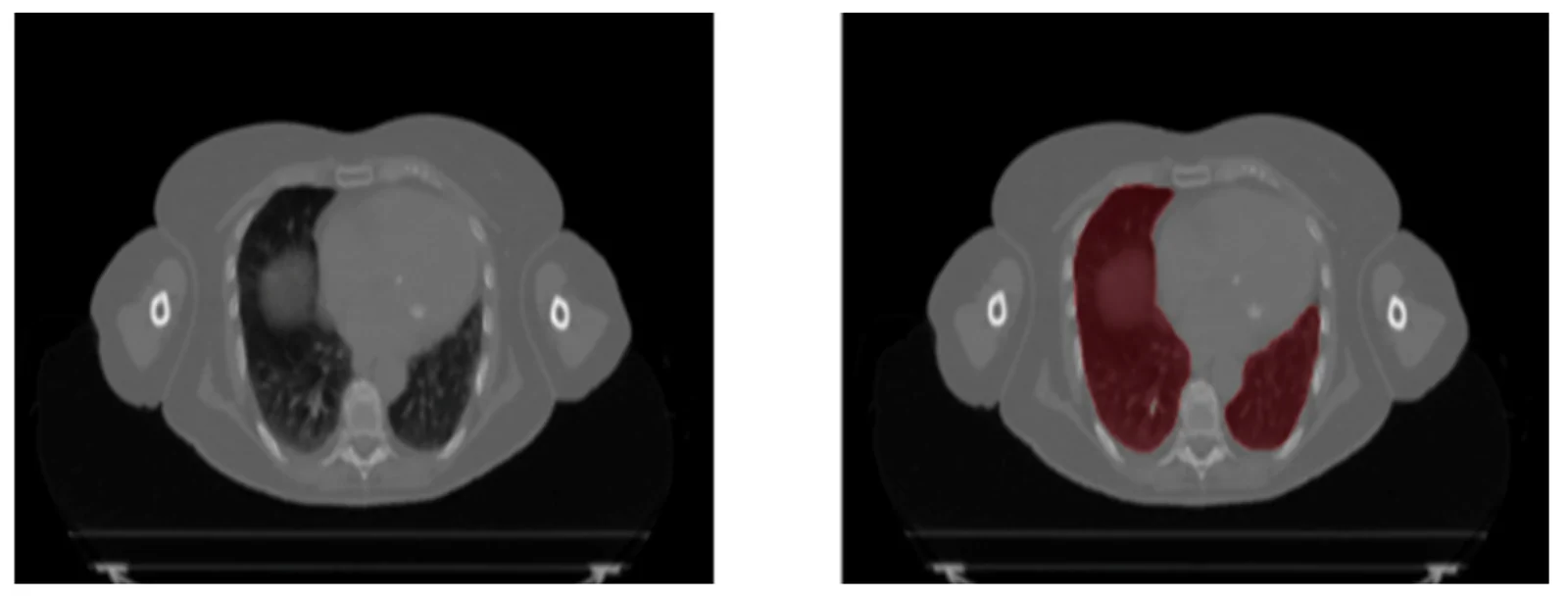
Visualization of the Average Intensity Projection (AIP) CT scan serving as the spatial reference frame, shown alongside the lung segmentation highlighted in red which is displayed as a Region of Interest (ROI) overlay on the CT image.

**Figure 3:**
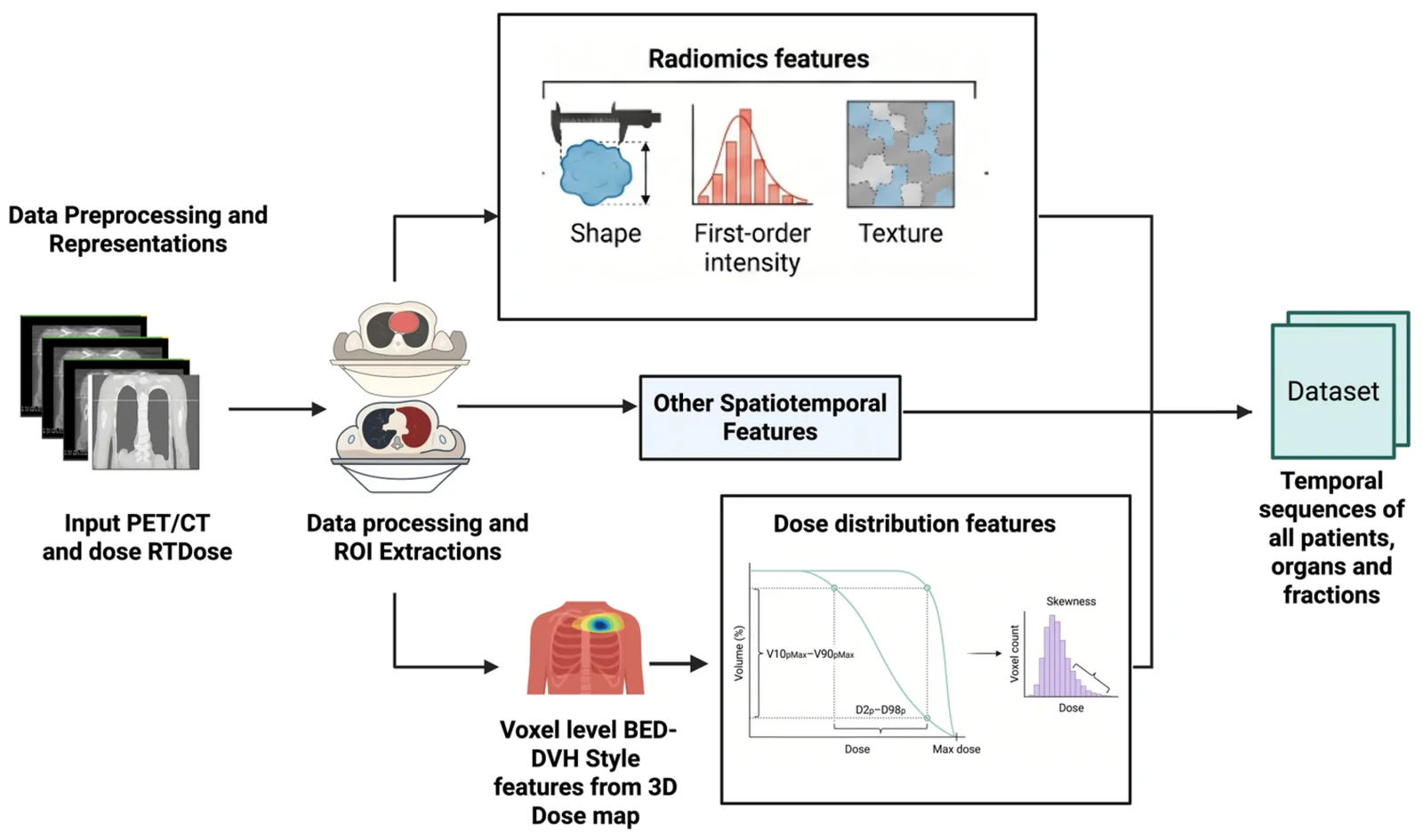
Multimodal feature extraction workflow for COMPASS. Per-fraction PET/CT and RT dose distributions are preprocessed and co-registered, with organ-specific ROIs delineated from RTSTRUCT contours. Feature extraction proceeds along three parallel branches: an imaging branch yielding shape, first-order intensity, and texture radiomics; a dose branch converting voxel-wise physical dose to BED/EQD2 and extracting DVH-style and dose-distribution features; and a spatiotemporal branch capturing plan quality, temporal kinetics, and geometric descriptors. Outputs are concatenated per patient–organ–fraction observation and assembled into the longitudinal dataset for temporal modeling.

**Figure 4:**
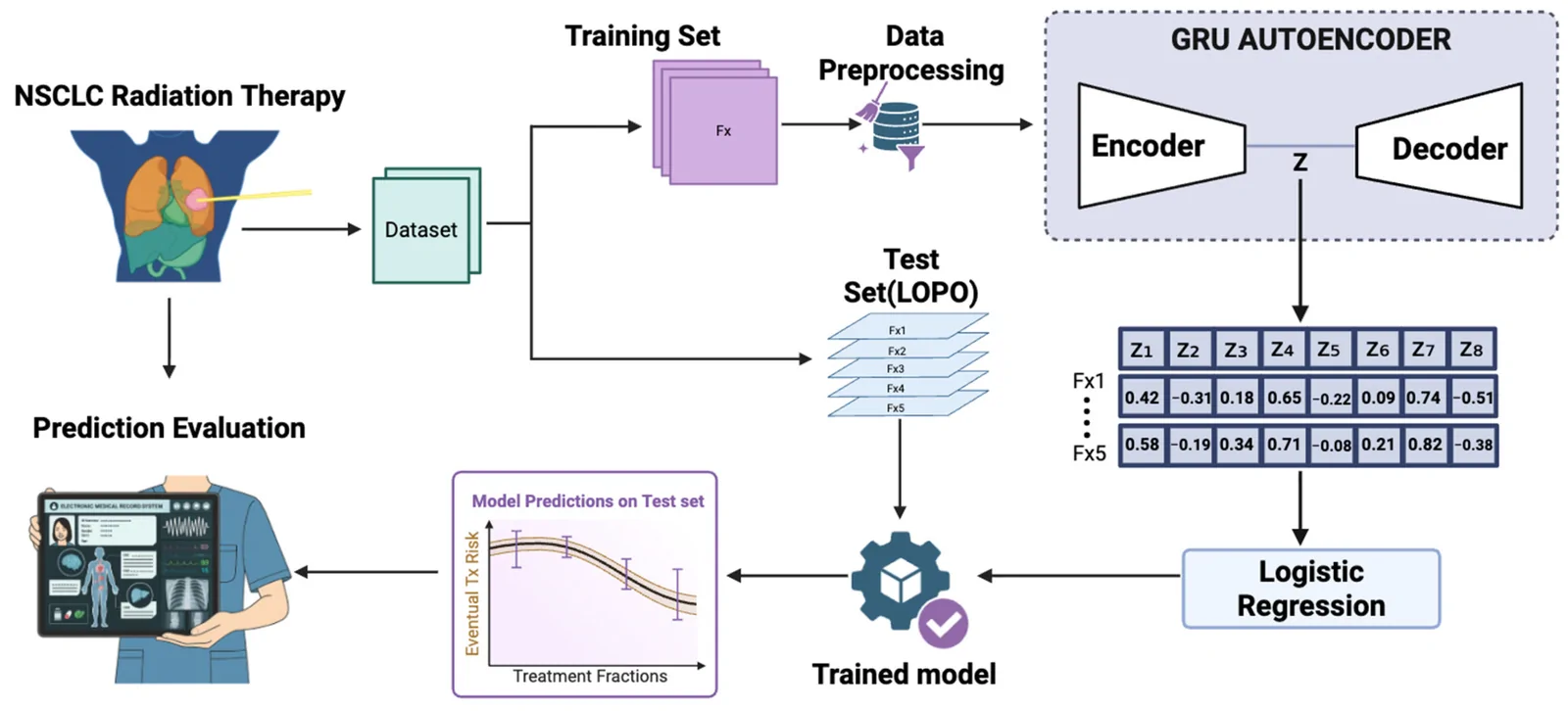
Temporal sequence modeling architecture and inference phase. The multimodal longitudinal dataset is partitioned into training and test sets using leave-one-patient-out (LOPO) cross-validation. During training, per-fraction sequences from the training patients are preprocessed and passed through a GRU autoencoder that learns 8-dimensional latent embeddings (Z), on which a logistic regression classifier is fit. During inference, the held-out test patient’s sequences pass through the frozen autoencoder and trained classifier using preprocessing parameters learned exclusively on the training set. The classifier produces per-fraction toxicity risk trajectories evaluated against observed outcomes.

**Figure 5:**
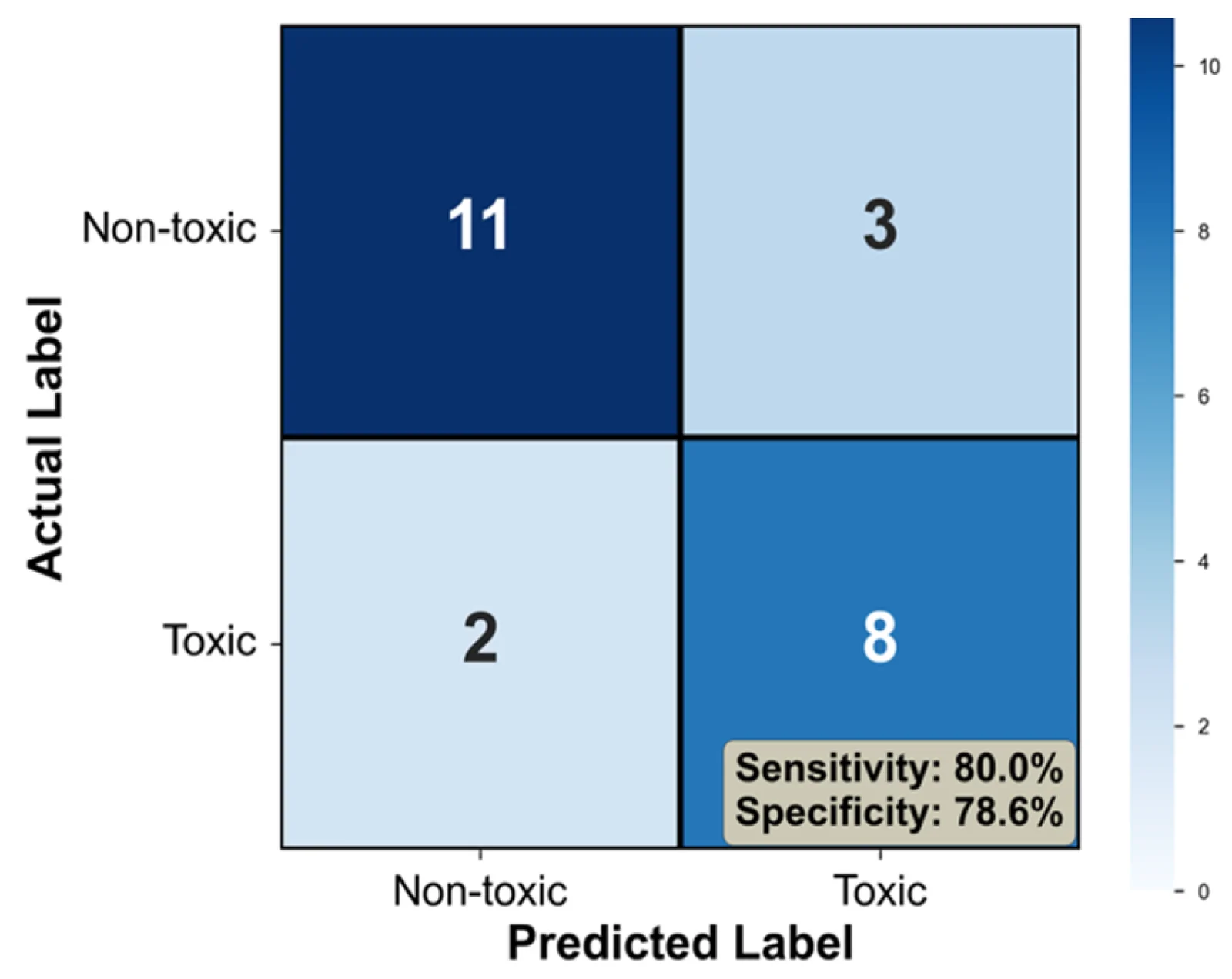
Aggregated confusion matrix for toxicity prediction. Confusion matrix summarizing model performance across all eight leave-one-patient-out (LOPO) folds for binary toxicity classification (toxic vs. non-toxic) on 24 patient–organ test cases. The model yields 11 true negatives, 8 true positives, 3 false positives, and 2 false negatives, corresponding to an apparent sensitivity of 80.0% and a specificity of 78.6%.

**Figure 6:**
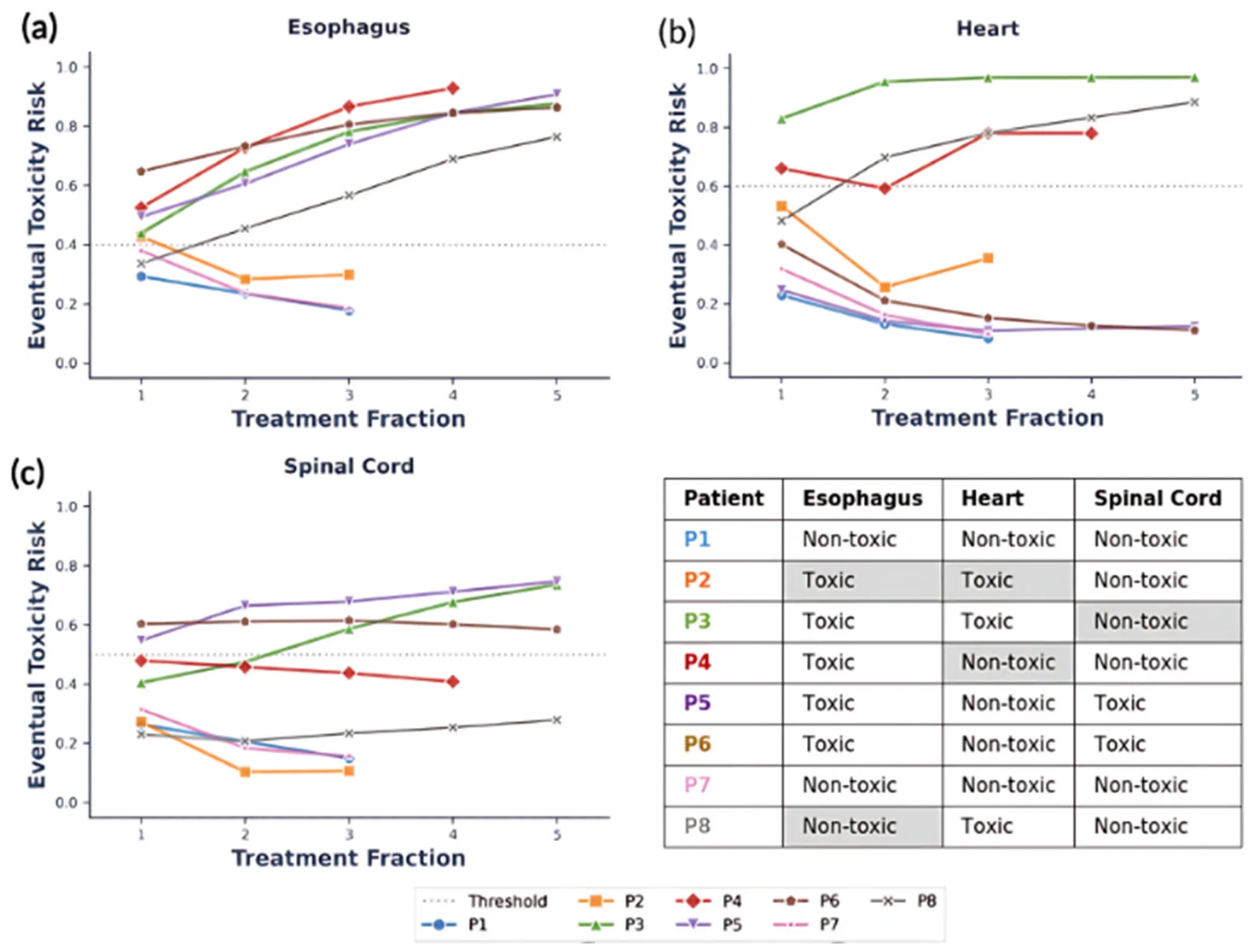
Temporal trajectories of predicted toxicity risk. Per-fraction model-predicted probabilities of eventual CTCAE ≥ 1 toxicity for the (**a**) esophagus, (**b**) heart, and (**c**) spinal cord across treatment fractions. Each line represents an individual patient–organ trajectory. Trajectories corresponding to organs that ultimately developed toxicity demonstrate progressive risk escalation, whereas non-toxic trajectories remain consistently low, highlighting the dynamic nature of longitudinal risk assessment. The table indicates the ground truth with the highlighted regions accounting for misclassified samples.

**Figure 7:**
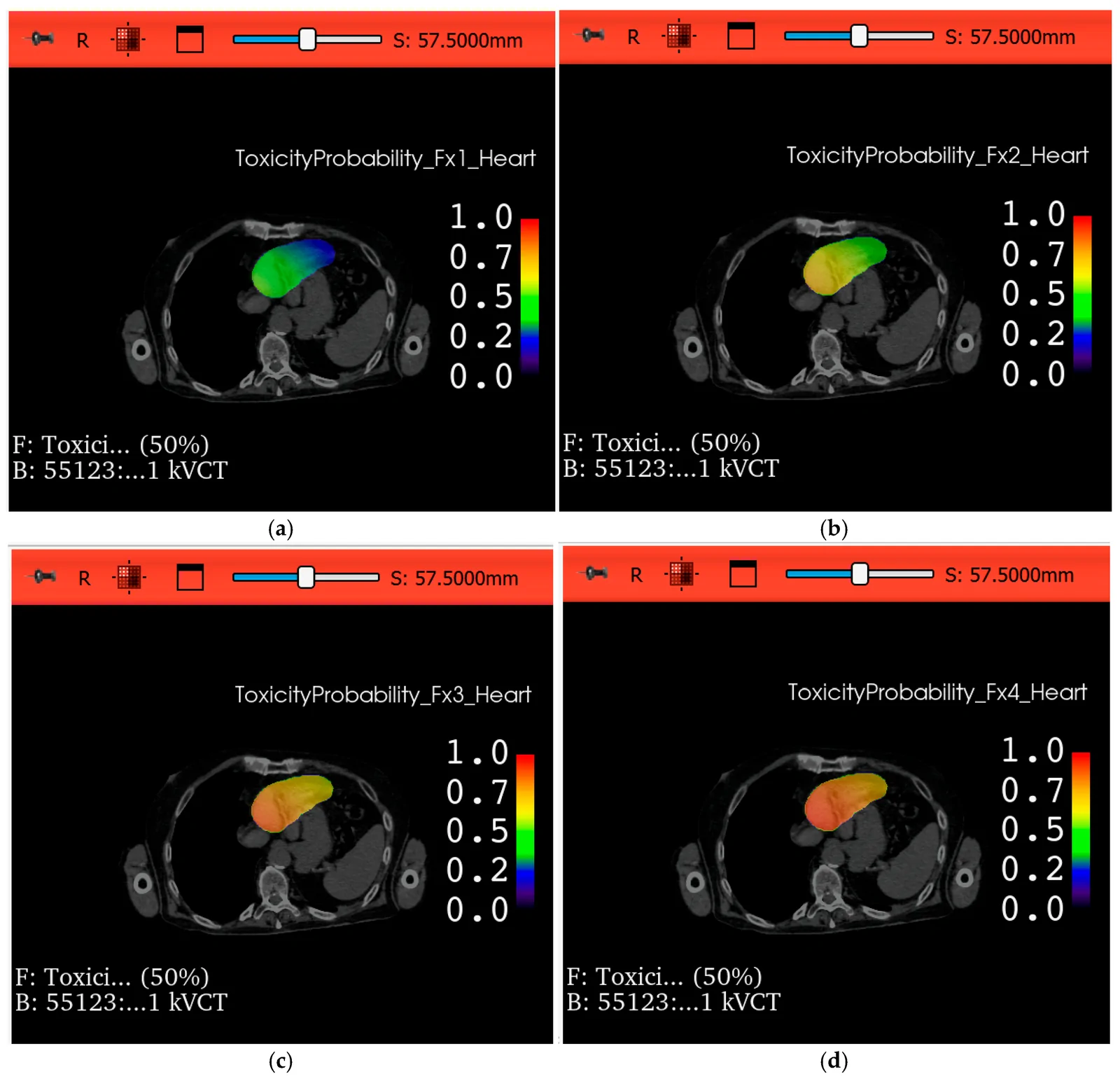
Per-voxel toxicity probability heatmaps across fractions. Representative axial CT slices of the heart across five treatment fractions, (**a**) Fraction 1, (**b**) Fraction 2, (**c**) Fraction 3, (**d**) Fraction 4, illustrating voxel-wise toxicity probability maps. Voxel-wise probabilities were computed from a physics-based exponential dose–response formula applied to cumulative voxel dose using a fixed, organ-specific dose threshold, independent of the organ-level trained GRU autoencoder/logistic regression model. Color overlays (scale 0.0–1.0) depict spatially resolved toxicity risk, enabling localization of emerging biological vulnerability hotspots within the organ during treatment.

**Table 1:** Summary of the 73 raw multimodal features engineered per patient–organ–fraction observation categorized by modality along with their implications for the COMPASS framework.

Category	No. of Features	Representative Metrics	Physics–Biological Interpretation
Dosimetric (DVH)	10	Dmax, Dmean, D5, D50, D95, D2cc, V5, V10, V20	Overall organ exposure; cumulative dose burden
Dose Distribution (Dosiomics)	25	D2p–D98p, V10pMax–V90pMax, skewness	Spatial dose heterogeneity; hotspot detection
Plan Quality Indices	4	Dose homogeneity, conformity, gradient index	Dose distribution quality; spatial falloff
Temporal Kinetics	2	ΔEQD2 per fraction, interfraction time	Dose accumulation rate; treatment recovery
Geometric	2	Organ volume, fractional hotspot volume	Morphology; dose localization
CT Intensity (Radiomics)	15	Mean, std, percentiles, skewness, entropy	Tissue density; anatomical heterogeneity
PET Intensity (Radiomics)	15	Mean, std, percentiles, skewness, entropy	Heterogeneity; spatial variation in PET uptake intensity

## Data Availability

The data presented in this study is available on request from the corresponding author. The data is not publicly available due to privacy issues.
